# Relationship between homocysteine level and diabetic retinopathy: a systematic review and meta-analysis

**DOI:** 10.1186/s13000-014-0167-y

**Published:** 2014-09-26

**Authors:** Chong Xu, Yan Wu, Guodong Liu, Xiaoqiang Liu, Fang Wang, Jing Yu

**Affiliations:** Department of Opthalmology, Nanshan Maternity & child healthcare hospital of Shenzhen, Shenzhen, China; Department of Ophthalmology, Shanghai Tenth People’s Hospital, Affiliate of Tongji University School of Medicine, Shanghai, 200072 China; Department of First Clinical Medical College, Nanjing Medical University, Nanjing, Jiangsu China

**Keywords:** Hyperhomocysteinemia, Homocysteine, Diabetic retinopathy (DR)

## Abstract

**Background:**

The relationship between homocysteine (Hcy) and diabetic retinopathy (DR) remains unclear to date. Therefore, a systematic review and meta-analysis was performed on the relationship between Hcy level and DR.

**Methods:**

Studies were identified by searching PubMed, Embase, and Web of Science databases until 5 May, 2014.

**Results:**

A total of 31 studies involving 6,394 participants were included in the meta-analysis. After pooling the data from each included study, the blood Hcy concentration in the DR group was observed to be higher than that in the control group [WMD = 2.55; 95% confidence interval (CI), 1.70–3.40], and diabetes mellitus (DM) patients with hyperhomocysteinemia were at a risk for DR [odds ratio (OR) = 1.93; 95% CI, 1.46–2.53]. Considering the different DM types, hyperhomocysteinemia in T1DM (OR = 1.83, 95% CI, 1.28–2.62) was associated with DR rather than in T2DM (OR = 1.59, 95% CI, 0.72–3.51). Considerable statistical heterogeneity in the overall summary estimates was partly explained by the geographical differences.

**Conclusions:**

Results from this current meta-analysis indicate that hyperhomocysteinemia is a risk factor for DR, especially proliferative DR. Differences between geographical regions were observed in the relationship between hyperhomocysteinemia with T1DM risk. Given the heterogeneous results, the relationship between high Hcy and DR needs further investigation.

**Virtual Slides:**

The virtual slide(s) for this article can be found here: http://www.diagnosticpathology.diagnomx.eu/vs/13000_2014_167

## Background

Diabetic retinopathy (DR), a common complication of diabetes, is the leading cause of low vision and blindness worldwide [1]. To date, no effective treatment has been found. Therefore, finding new risk factors and biomarkers to prevent the progression of DR is important. A number of risk factors are associated with the incidence of DR, such as blood pressure, blood glucose, glycosylated hemoglobin, blood urea nitrogen, creatinine, and β2-microglobulin. However, the risk factors for DR are complicated by many aspects.

Homocysteine (Hcy) is a sulfur-containing amino acid formed by the demethylation of methionine; it is an emerging risk factor for diabetic nephropathy and cardiovascular disease that has gradually elicited the interest of researchers [2]. These researchers found that the level of Hcy is related to DR, especially proliferative DR [3,4].

However, studies on the relationship between Hcy and DR have reported inconsistent results, and the role of Hcy in the development of DR is not clearly elucidated. Several studies have shown relationships between the blood Hcy level and the prevalence of DR [5,6]. Plasma total Hcy concentration may be a useful biomarker for increased risk of DR in people with type 2 diabetes mellitus (T2DM) [7] and a complication risk indicator in type 1 diabetes mellitus (T1DM) [3]. Moderate hyperhomocysteinemia has been described in T1DM, which may be linked between the conditions of mutation in the methylenetetrahydrofolate reductase (MTHFR) gene [8,9]. On the other hand, other studies did not find any relationship between plasma Hcy and different grades of DR on adults with T1DM, except when concomitant nephropathy existed [10,11].

Given the absence of uniform research standards and inconsistency in research results, a meta-analysis of prospective studies on the relationship between Hcy and DR was conducted.

This meta-analysis mainly focused on the following objectives: (1) to review and summarize the epidemiologic evidence on the relationship between hyperhomocysteinemia and risk of DR and (2) to quantify the mechanism of hyperhomocysteinemia and DM relationship according to study characteristics.

## Methods

### Search strategy

The meta-analysis was conducted according to the Preferred Reporting Items for Systematic Reviews and Meta-Analysis guidelines [12].

### Study selection

We searched PubMed, Embase, and Web of Science to retrieve related studies published before May, 2014. Literature search was performed using keywords “homocysteine,” “hyperhomocysteinemia,” “Hcy,” and “homocysteic acid” in combination with “diabetic retinopathy” and “diabetic retinitis.” Moreover, the citations of related articles were detected for additional publications. No language or date limitations were set. Corresponding authors of the retrieved articles were contacted for additional information.

### Inclusion criteria

Studies that met the following inclusion criteria were included in this meta-analysis: (1) comparative studies; (2) reported either the prevalence in hyperhomocysteinemia patients and the controls or the concentrations of Hcy in DR and the controls; and (3) provided the value of mean with 95% confidence intervals (CIs), standard errors or sufficient data to calculate them.

### Exclusion criteria

Studies that met the following inclusion criteria were excluded in this meta-analysis: (1) not for DR research; (2) studies with small sample size (less than 40 cases or control group) and low quality (NOS, less than 6 points) (3) duplicate of previous publication; and articles without available data; (4) the plasma Hcy levels of studies are influenced seriously by pathological factors.

### Data extraction

Data were independently extracted from each included article by two reviewers (C. Xu and Y. Wu), and all disagreements on eligibility during the extraction were discussed and resolved by the reviewers. The first author, country, year of publication, number of both case and control groups, blood components used for the test, DM type, definition of hyperhomocysteinemia, and matching factors were extracted.

### Assessment of methodology quality

The Newcastle–Ottawa scale (NOS) [13] was adopted for assessing the quality of the included retrospective observational studies. The maximal NOS score was 9 points, and studies ≥6 points were considered to be of relatively higher quality.

### Statistical analysis

This meta-analysis was conducted using the Stata software package (version 11.0; Stata Corp., College Station, TX). A random-effect method was conducted to pool the results together [14]. The pooled odds ratios (ORs) and weighted mean difference (WMD) were measured with 95% CIs. Both the ORs and WMD were considered statistically significant. The subgroup analyses were performed based on DM type, site, blood component, and study design. Both the χ^2^ and I^2^ tests were used to evaluate the statistical heterogeneity among studies, and the heterogeneity was considered statistical when P < 0.1 and I^2^ ≥ 50%. Meta-regression method was used to evaluate the source of heterogeneity.

The sensitivity analysis evaluated the robustness of the results in this study. Two different methods were performed to conduct this analysis. First, the articles were excluded one by one, and then a meta-analysis was again conducted. Second, studies with small sample size (less than 40 participants in either case or control group) and low quality (NOS, less than 6 points) were excluded. To evaluate the potential publication bias, both the funnel plot and the Egger test were performed [15,16].

## Results

### Literature search

Of the 299 articles (72 from PubMed, 147 from Embase, and 80 from Web of Science) identified from database searches, 132 duplicates were excluded. After reviewing the titles and abstract of the 167 remaining articles, 114 articles were excluded and 53 full-text articles were assessed for eligibility. A total of 24 articles (four from duplicated data sources and 20 without available data) were excluded. Moreover, two studies were identified from reference lists. Thirty-one articles were included in this meta-analysis [2-11,17-37], in which nine reported the relationship between hyperhomocysteinemia and risk of DR and 27 reported the concentrations in DR and NDR (six studies reported both results).

### Characteristics and baseline of the included studies

Up to 6394 individuals were included in this meta-analysis. A total of 609 cases and 238 controls comprised the comparison between the risk of DR in hyperhomocysteinemia and control groups, and 2070 cases and 3477 controls comprised the comparison between the concentrations of Hcy in DR and NDR groups. The definitions of hyperhomocysteinemia were different in each study, and four of nine studies used 15 μmol/l in the plasma as cut-off. Although the definitions were not the same, no significant differences existed in the definitions. Both plasma and serum were used for the measurement of the concentrations of Hcy in most studies. All of the included articles were retrospective case–control studies. Of the 31 included studies, 4 were conducted in the United States, 11 in Asia, 14 in Europe, and 2 in Oceania. Age and gender were matched in most of the studies. Several studies displayed other comparable factors. Tables 1 and 2 show the characteristics and baselines of the included studies.

**Table 1:** **Characteristics of the studies on the relationship between hyperhomocysyeinemia and risk of diabetic retinopathy**

**Source**	**Country**	**No. of case/control**	**DM Type**	**Blood**	**Exposure definition**	**Study quality** ^**†**^	**Matching/comparable** ^*^
Golbahar et al. 2008 [17]	Bahrain	124/130	2	Plasma	F: Hcy > 15 μmol/l; M: Hcy > 12 μmol/l	8	1, 2, 3, 4, 7, 12, 13
Vaccaro et al , 1997 [8]	Japan	11/14	1	Plasma	≥10 μmol/	6	1, 2, 6, 8, 9, 13
Hofmann et al., 1998 [18]	France	26/49	1	Plasma	Preload > 15.8 μmol/l or Postload >31.1 μmol/l	8	1, 2, 3, 4, 6, 7, 9
Hoogeveen et al., 2000 [5]	Netherlands	534/91	2	serum	>16 μmol/l	7	1, 2, 7, 8, 9
Buysschaert et al., 2000 [19]	Belgium	38/84	1	Plasma	>15 μmol/l	7	1, 2, 3, 5, 6, 10, 11, 12
Agullo-Ortuno et al., 2002 [3]	Spain	18/71	1, 2	Plasma	F >13.9 μmol/l, M >15.6 μmol/l	5	9, 10, 11, 12
Goldstein et al., 2004 [4]	Israel	84/251	NA	Plasma	>15 μmol/l	7	1, 6, 14
de Luis et al., 2005 [20]	Spain	22/133	2	Plasma	≥ 15 μmol/l	6	1, 6, 7, 8, 9
Satyanarayana et al., 2011 [21]	US	141/159	2	Plasma	> 12 μmol/l	7	1, 5, 9, 11

NA: not available; DM: diabetes mellitus; F: female; M: male; Hcy: homocysteine.

^†^Study quality is evaluated by Newcastle-Ottowa Scale (1–9 stars).

*The matching factors are: (1) age, (2) gender, (3) diabetes duration, (4) diabetes control, (5) hypoglycemia medication, (6) blood pressure, (7) BMI, (8) creatinine, (9) plasma cholesterol, (10) triglyceride, (11) low densitv lipoprotein, (12) high densitv lipoprotein, (13) smoking status, (14) other complications.

**Table 2:** **Characteristics of the studies on the comparison between the concentrations of homocysteine in the DR and NDR group**

**Source**	**Country**	**No. of case/control**	**DM Type**	**Case group/control group**	**Blood**	**Study quaility** ^**†**^	Matching/comparable*
Chen, 2010 [22]	China	88/95	2	DR/normal or DM	Plasma	6	1 2
Golbahar et al. , 2008 [17]	Bahrain	124/130	2	DR/DM	Plasma	8	1, 2, 3, 7, 8, 9, 10, 11, 12, 13
Lim et al., 2012 [23]	Malaysia	20/21	2	PDR/Normal	Plasma	5	1, 2
Hultberg et al., 1991 [24]	Sweden.	67/46	1	DR/Normal	Plasma	6	1, 2, 6, 13
Chico et al., 1998 [2]	Spain	71/62	1,2	DR/DM	Plasma	8	1, 2, 7, 14
Smulders et al., 1999 [25]	Netherlands	66/71	2	DR/DM	Plasma	7	2
Stabler et al., 1999 [26]	US	218/223	2	DR/DM	Plasma	6	1, 2, 14
Agardh et al., 2000 [10]	Sweden.	25/24	1	DR/DM	Plasma	7	1, 2, 3, 5, 6, 7
Vaccaro et al., 2000 [9]	Italy	30/34	NA	DR/DM	Plasma	7	1, 2, 6, 13
Chiarelli et al., 2000 [27]	US	141/117	1	DR/Normal or DM	Plasma	8	1, 2, 3, 5
Buysschaert et al., 2001 [28]	France	24/47	1	DR/DM	Plasma	5	1, 2, 5
Agullo-Ortuno et al., 2002 [3]	Spain	18/71	1	DR/DM	Plasma	6	9, 10, 11, 12
Yang et al., 2002 [29]	China	16/58	2	DR/Normal or DM	Plasma	7	1, 2, 6, 7
Abdella et al., 2002 [30]	Kuwait	145/213	2	DR/DM	Plasma	6	1, 3, 5, 13
Looker et al., 2003 [31]	US	102/279	2	DR/DM	Serum	8	2, 4, 5, 6, 13
Saeed et al., 2004 [11]	UK	25/23	1	NPDR/DM	Serum	7	1, 2, 3, 6, 7, 13
Goldstein et al., 2004 [4]	Israel	117/218	NA	DR/Normal or DM	Plasma	6	1, 6, 14
de Luis et al., 2005 [20]	Spain	22/133	2	DR/DM	Plasma	7	1, 6, 7, 8, 9
García-Unzueta et al., 2005 [32]	Spain	38/117	1	DR/DM	Serum	4	NA
Yücel I, 2004[33]	Turkey	20/30	2	PDR/Normal	Plasma	4	1, 2
Huang et al., 2006 [6]	China	91/370	2	DR/Normal or DM	Plasma	7	1, 2, 9, 11
Brazionis et al., 2008 [7]	Australia	48/120	2	DR/DM	Plasma	8	1, 2, 4, 6, 10, 12, 13
Aydin E, 2008 [34]	Turkey	39/35	2	DR/Normal or DM	Plasma	4	1, 2
Aydemir et al., 2008 [35]	Turkey	20/12	2	PDR/Normal	Plasma	5	1
Nguyen et al., 2009 [36]	Australia	278/743	NA	DR/DM	Plasma	7	1, 2, 7, 9, 10, 13
Satyanarayana et al., 2011 [21]	US	150/150	2	DR/Normal or DM	Plasma	7	1, 5, 9, 11
Cho, 2011 [37]	Korea	67/35	2	DR/DM	Plasma	6	1, 6, 7, 11, 12

NA: not available; DM: diabetes mellitus; DR: diabetic retinopathy; PDR: proliferative diabetic retinopathy.

^†^Study quality is evaluated by Newcastle-Ottowa Scale (1–9 stars).

*The matching factors are: (1) age, (2) gender, (3) diabetes duration, (4) diabetes control, (5) hypoglycemia medication, (6) blood pressure, (7) BMI, (8) creatinine, (9) plasma cholesterol, (10) triglyceride, (11) low densitv lipoprotein, (12) high densitv lipoprotein, (13) smoking status, (14) other complications.

### Quality assessment

NOS was used to evaluate the methodological quality of each study because all of the included articles were retrospective case–control studies. Among the 31 studies, six were less than 6 points, and the mean NOS score was 6.45 ± 1.21 points. The lowest and the highest scales were 4 points in two studies and 8 points in six studies, respectively.

### Efficacy analysis

#### Main results

**Figure 1:**
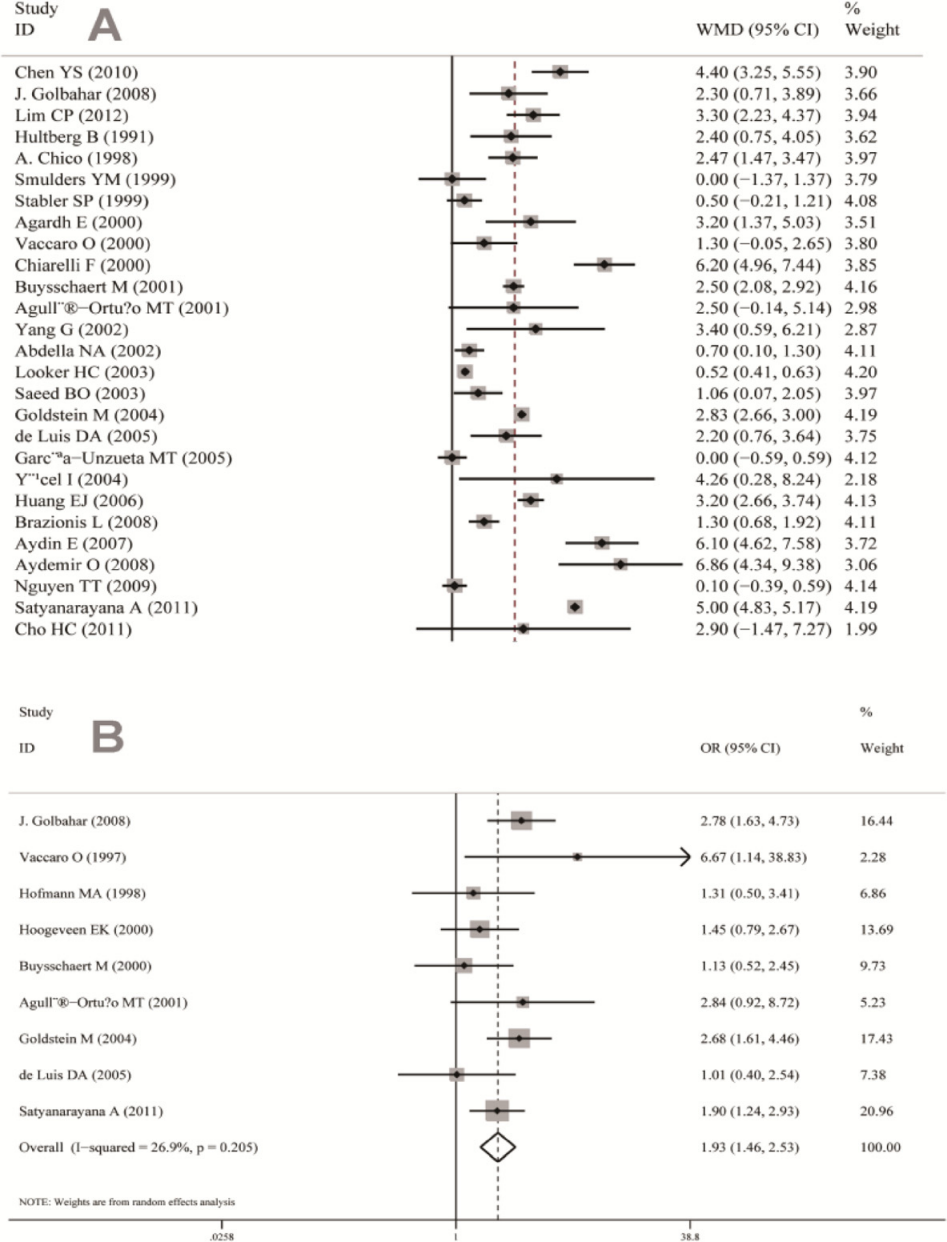
**Forest plot of relationship between homocysteine level and diabetic retinopathy.** **(A)** The concentration of Hcy was higher in DR groups than the control group (WMD, 2.55; 95% CI, 1.70 to3.40) **(B)** Diabetes mellitus (DM) patients with hyperhomocysteinemia were at a risk for DR [odds ratio (OR) = 1.93; 95% CI, 1.46–2.53].

Figure 1 presents the main results of this current meta-analysis. Patients with hyperhomocysteinemia were at higher risk of developing DR compared with the control group (OR, 1.93; 95% CI, 1.46 to 2.53). The interstudy heterogeneity was not significant (I^2^ = 26.9%, P = 0.205). A higher concentration of Hcy in the blood was observed in the DR group (WMD, 2.55; 95% CI, 1.70 to 3.40). However, a significant heterogeneity was also detected in the comparison (I^2^ = 98.8%, P < 0.001).

#### Subgroup analysis

Subgroup analysis was conducted by DM type, geographic site, blood components, and study designs (Table 3). In the different DM types, T2DM patients with hyperhomocysteinemia did not show higher risk of developing DR (OR, 1.59; 95% CI, 0.72 to 3.51). However, in both T1DM and mixed-type DM groups, higher incidence rates of DR were detected in the hyperhomocysteinemia group (T1DM: OR, 1.83; 95% CI, 1.28 to 2.62; Mixed: OR, 1.93; 95% CI, 1.46 to 2.53). In all of the three different DM type groups, the concentration of Hcy was higher in DR groups (T1DM: WMD, 2.5; 95% CI, 1.04 to 3.97; T2DM: WMD, 2.85; 95% CI, 1.45 to 4.25; Mixed: WMD, 1.68; 95% CI, 0.01 to 3.35).

**Table 3:** **Relationship between hyperhomocysteinemia and risk of diabetic retinopathy, and the concentrations of hcy between the DR and NDR groups by subgroup analysis**

	**Hyperhomocysyeinemia and risk of diabetic retinopathy**					**Homocysteine in DR and NDR**				
**Factor**	**No. of studies**	**OR**	**95%** **CI**	**Heterogeneity**		**No. of studies**	**WMD**	**95%** **CI**	**Heterogeneity**	
				***P***	**I** ^**2**^ **(%)**				***P***	**I** ^**2**^ **(%)**
DM type										
T1D	3	1.83	1.28 to 2.62	0.21	33.6	7	2.5	1.04 to 3.97	0.00	94.0
T2D	4	1.59	0.72 to 3.51	0.19	39.7	16	2.85	1.45 to 4.25	0.00	99.2
Mixed	2	1.93	1.46 to 2.53	0.93	0.0	4	1.68	0.01 to 3.35	0.00	97.2
Site										
Unitd Staes	1	1.9	1.24 to 2.93	-	-	4	3.03	−0.02 to 6.08	0.00	85.0
Asia	4	2.36	1.46 to 3.81	0.15	44.3	11	3.43	2.59 to 4.28	0.00	88.6
Europe	4	1.45	0.95 to 2.20	0.57	0.0	10	1.68	0.83 to 2.52	0.00	85.0
Oceania	-	-	-	-	-	2	0.68	−0.49 to 6.08	0.00	99.8
Blood										
Plasma	8	2.01	1.49 to 2.72	0.20	29.0	24	2.81	2.04 to 3.57	0.00	97.3
Serum	1	1.45	0.79 to 2.67	-	-	3	0.45	0.03 to 0.87	0.13	51.5
Study design										
DR vs normal	3	3.71	0.94 to 14.72	0.0	90.9	11	5.21	3.50 to 6.92	0.00	99.2
DR vs DM	9	2.02	1.52 to 2.68	0.33	12.1	22	2.34	1.62 to 3.05	0.00	97.6
NPDR vs control	2	2.66	1.74 to 4.06	0.88	0.0	10	1.8	0.89 to 2.71	0.00	96.4
PDR vs control	2	2.84	1.75 to 4.61	0.98	0.0	12	4.53	3.16 to 5.90	0.00	91.6

-: no data; DM: diabetes mellitus; T1D: type 1 diabetes mellitus; T2D: type 2 diabetes mellitus; DR: diabetic retinopathy; PDR: proliferative diabetic retinopathy; NPDR: nonproliferative diabetic retinopathy; hcy: homocysteinemia.

In the United States and Asia, a higher incidence rate of DR was detected in the hyperhomocysteinemia group (US: OR, 1.9; 95% CI, 1.24 to 2.93; Asia: OR, 2.36; 95% CI, 1.46 to 3.81). However, in Europe, the incidence rate of DR was not higher in the hyperhomocysteinemia group compared with the control group (OR, 1.45; 95% CI, 0.95 to 2.20). In Asia and Europe, the concentrations of Hcy were higher in the DR group compared with the control group (Asia: WMD, 3.43; 95% CI, 2.59 to 4.28; Europe: WMD, 1.68; 95% CI, 0.83 to 2.52). However, the concentrations of Hcy in the DR groups were not significantly higher in the US and Oceania (US: WMD, 3.03; 95% CI, −0.02 to 6.08; Oceania: OR, 0.68; 95% CI, −0.49 to 6.08).

Four different study designs, namely, DR vs. normal, DR vs. DM, non-proliferative diabetic retinopathy (NPDR) vs. control, and PDR vs. control, were used in the subgroup analysis. A higher incidence rate in hyperhomocysteinemia was detected in all of the subgroups, and a higher concentration of Hcy was detected in the DR group. No inverse results were detected in the subgroup analysis compared with the main results.

The incidence of DR was compared in the two groups (high Hcy and control). Only the subgroup analysis between the DR and the normal group was significantly heterogeneous (I^2^ = 90.9, P < 0.01). However, most subgroup analyses exhibited significant heterogeneity compared with the Hcy concentrations in DR and NDR. No significant heterogeneity was observed between the DR and the NDR groups, whereas the comparison of Hcy serum was significantly heterogeneous (I^2^ = 51.5, P = 0.13).

#### Heterogeneity, sensitivity analysis, and publication bias

Several outcomes were heterogeneous in this meta-analysis, and the subgroup analysis did not succeed in detecting the source of heterogeneity. In addition, meta-regression was conducted, and no sources of heterogeneity were detected.

After dropping each study one by one from the included studies, no significant different results were observed. A meta-analysis was conducted after excluding the studies with small sample size (less than 40 participants in either case or control group) and low quality (NOS, less than 6 points). After excluding five studies, a significant relationship was observed between hyperhomocysteinemia and risk of DR (OR, 2.16; 95%, 1.63 to 2.85). Moreover, after excluding 12 studies, the concentration of Hcy in the DR group was still higher than that in the control group (WMD, 2.16; 95% CI, 1.12 to 3.40).

**Figure 2:**
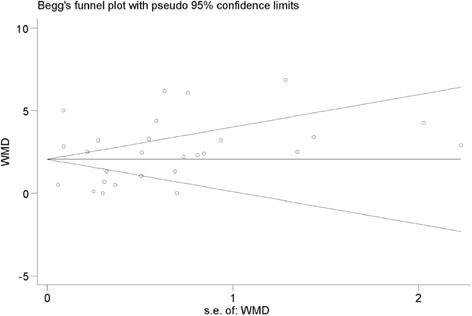
**Funnel plot of the included studies.** Funnel plot of the WMD vs the standard error of the log WMD for studies evaluating homocysteine level and diabetic retinopathy.

Both the funnel plot (Figure 2) and the Egger test showed that no potential publication bias existed in the comparison between the incidence rate of the hyperhomocysteinemia group and the control (P = 0.07) group and the comparison between the concentration of Hcy in DR and control group (P = 0.63).

## Discussion and conclusions

A large number of studies focused on the relationship between Hcy and retinopathy, but their results were inconsistent. Studies on T1DM reported positive relationships between DR and Hcy [38,39]. Hcy significantly increased in DM patients, and Hcy may be related to longer diabetic duration and DR [6]. Elevated Hcy was also related to increased risk for proliferative retinopathy [31,34]. A high prevalence of retinopathy was found in patients with T2DM who had fasting Hcy concentrations greater than 15 μmol/l [3,5]. Hcy may be a predictor of retinopathy in T2DM [7,9,37].

In some studies, no significant correlations existed between plasma Hcy and different grades of DR in patients with T1DM, except when a concomitant nephropathy was present [10,11,30,36]. Hyperhomocysteinemia is probably caused by early nephropathy rather than a result of the diabetic process itself [2,11,40]. The reasons for these inconsistencies may be due, in part, to differences in the study sample (e.g., groups with different diabetic duration), study design, definitions of DR (e.g., clinical versus photograph grading), and failure in some studies to make adequate adjustments for traditional risk factors.

A comprehensive and quantitative summary was lacking on the relationship between Hcy and DR. Therefore, a meta-analysis was conducted to assess the relationship between Hcy and DR. Based on the meta-analysis of the DR groups compared with the normal or DM patients, Hcy concentration in the blood was higher than in the control. These results are the same as those of the PDR groups compared with the control, especially in Asia and Europe. No significant differences were observed in Australia and USA. These findings may be associated with research heterogeneity or the district biases. The different research methods and the sensitivity to detect retinopathy by fundus photography or misclassification of retinopathy may result in district bias.

Plasma Hcy levels are influenced by environmental and genetic factors [41], as well as by age, sex, duration of diabetes, smoking habits, body mass index, metabolic control, creatinine clearance, impaired renal function, vitamin status, and blood pressure [11,21,28,42]. The kidney has an important function in maintaining Hcy levels. The enzyme MTHFR is active in the kidney, and impairment of renal function can lead to hyperhomocysteinemia [11].

Many studies excluded serious pathology state of research groups, and different research groups matched for these factors, which could influence plasma Hcy levels. Therefore, we believe that hyperhomocysteinemia may be a potential risk factor for DR patients [31].

A higher incidence of DR exists in diabetic patients with hyperhomocysteinemia. According to the different DM types, a significant difference in T1DM group is observed, whereas no significant difference is detected in T2DM group. Given all the differences, the incidence of DR in hyperhomocysteinemia cases is higher than in the control, except in Europe. Although most relationships are not significant, a high homocysteinemia is common in the relationship with the incidence of DR, especially with PDR (Table 3). To understand the significance of the results in Table 3, heterogeneity and subgroup analyses were analyzed. In the comparison between the incidence of DR in different levels of plasma Hcy groups, only the DR groups compared with normal groups were significantly heterogeneous (I^2^ = 90.9, P < 0.01). This result may due to the different study designs with different methodological qualities.

The pathogenesis of hyperhomocysteinemia in the development of DR may accelerate cell injury (including oxidative stress and impaired generation of nitric oxide) and proliferate smooth muscle cells to alter vasomotor activity [43,44]. Cell injury at the level of the retinal capillary may contribute to the development of DR [8,18,45,46]. The relationship between PDR and plasma Hcy levels may be related to C677T mutation in the gene coding for the MTHFR [8,9,17,47].

A few studies demonstrated that vitreous hyperhomocysteine is related to DR because of the loss of blood–retinal barrier permeability. This result may augment the diffusion of Hcy into the vitreous and the changes in vascular permeability [35,48]. Therefore, an elevated Hcy level in the vitreous may be important in the pathogenesis of PDR [23].

Given the numerous factors influencing the development and progression of DR, some of them remain unknown. We believe that hyperhomocysteinemia is another contributing factor to DR, especially to the severity of retinopathy independent of other risk factors [4].

**Figure 3:**
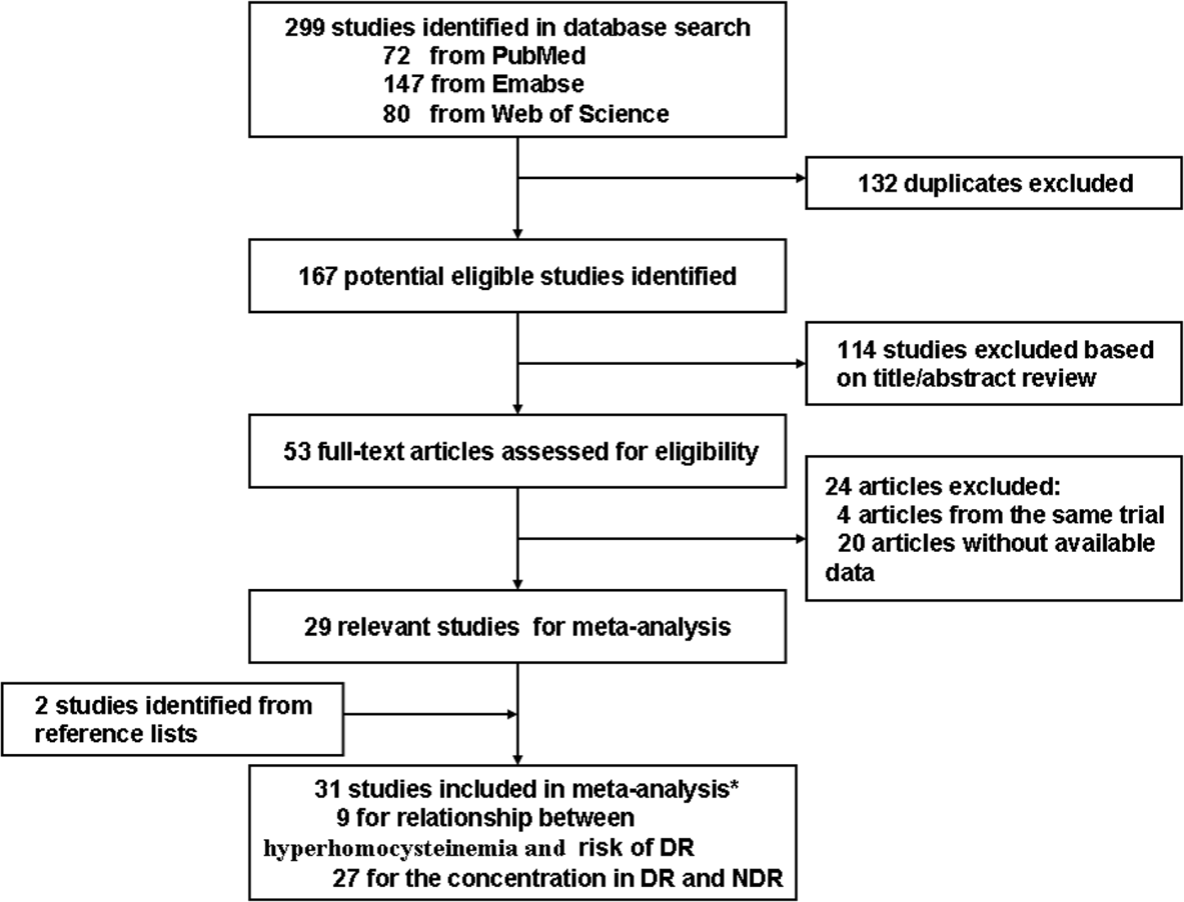
**Flow diagram showing the identification of relevant studies in the meta-analysis.** The initial 299 articles were identified and after 133 uplicates and 114 unrelated articles were excluded. A total of 53 full-text articles were assessed for eligibility. Twenty-four articles were excluded from and 2 articles from reviewing the reference lists of the related articles were included. In final, 31 articles were included in this meta-analysis.

The most significant conclusion that can be obtained from the data in Figures 1, 2 and 3 is the influence of concentration of plasma Hcy level on the incidence of DR and the progression of this microvascular complication of the retina, particularly in T1DM in Asia and the United States.

Some limitations of this meta-analysis are as follows. First, the included studies are not randomized controlled trials, and thus, may hold potential bias. Second, several outcomes were quite heterogeneous in this meta-analysis, and the subgroup analysis was not successful in detecting the source of heterogeneity. Moreover, we cannot obtain a possible threshold between the blood Hcy level and the separate degrees of DR because of the limited number of subjects with retinopathy and the different study designs.

The risk factors for DR are complicated by many aspects. Using Insulin may increase the risk of diabetic retinopathy [49], However, hyperhomocysteinemia correlates with insulin resistance and it could play a role in the higher risk of cardiovascular disease in obesity [50]. Diabetic retinopathy (DR) was earlier recognized as a vascular disease, but nowadays, it is considered as a neurovascular disorder. hyperhomocysteinemia plays important role in causation of retinal ganglionic cell apoptosis in diabetic patients [51]. Moreover, gene–gene and gene–environment interactions should also be considered in future analysis.PPARγ2 Pro12Ala polymorphism is not a risk factor for developing T2D diabetic nephropathy [52]. The association of plasma Hcy level and MTHFR gene polymorphism also be widely studied [53]. MTHFR polymorphism is shown to be associated with lipid metabolism in the elderly women [54].And this consistence to our study that Hcy level is related to MTHFR (coded by C667Tgene) polymorphism and may be the risk of diabetic complications.

All these meta-analysis are very significant to show the relationship between the different risk factors with diabetic retinopathy and other diabetic Complications. But our meta show the relationship between Hcy and different aspect of DR deeply.

Hcy may be a potential useful biomarker in assessing the microvascular risk in diabetes, and hyperhomocysteinemia is a potential risk factor for DR, especially PDR. This result may help DM patients in obtaining an instructive management to prevent the progression of DR because the plasma Hcy level can be substantially lowered with folic acid supplementation. High Hcy on DR will result in increasing attention to the study of gene C667T polymorphism and related prevention and treatment methods. In addition, considering the limitation of this study, larger studies should be considered and conducted to validate the current meta-analysis.

## Abbreviation

Hcy: Homocysteine
DR: Diabetic retinopathy
